# A novel chalcone derivative exerts anti-inflammatory and anti-oxidant effects after acute lung injury

**DOI:** 10.18632/aging.102288

**Published:** 2019-09-24

**Authors:** Yuting Lin, Man Zhang, Qingdi Lu, Jingwen Xie, Jianzhang Wu, Chengshui Chen

**Affiliations:** 1 Department of Pulmonary Medicine, The First Affiliated Hospital, Wenzhou Medical University, Wenzhou, Zhejiang 325006, China; 2 Department of Orthopedics, The Second Affiliated Hospital Zhejiang University School of Medicine, Zhejiang 325000, China; 3 Department of Pharmacy, Pharmacy School, Wenzhou Medical University, Wenzhou, Zhejiang 325006, China

**Keywords:** acute lung injury, chalcone derivatives, inflammation, oxidative stress

## Abstract

We explored the effects of compound 33, a synthetic chalcone derivative with antioxidant activity, on lipopolysaccharide (LPS)-induced acute lung injury (ALI). Compound 33, dexamethasone or vehicle was administered intragastrically to mice 6 h before intratracheal instillation of LPS. After 24 h, the effects of compound 33 on alveolar structural damage were evaluated by assessing lung morphology and the wet/dry weight ratio. Protein and proinflammatory cytokine levels and superoxide dismutase activity were also examined in the cell free supernatant of bronchoalveolar lavage fluid. Additionally, we investigated the anti-inflammatory and antioxidant activity of compound 33 *in vitro* and its effects on the MAPK/NF-κB and Nrf2/HO-1 pathways. Pretreatment with compound 33 prevented LPS-induced structural damage, tissue edema, protein exudation, and overproduction of proinflammatory mediators. The effects of compound 33 were similar to or greater in magnitude than those of the positive control, dexamethasone. Moreover, compound 33 exerted anti-inflammatory and antioxidant effects *in vitro* by inhibiting the MAPK/NF-κB pathway and activating the Nrf2/HO-1 pathway. Compound 33 may therefore be a promising candidate treatment for ALI.

## INTRODUCTION

Acute lung injury (ALI) manifests clinically as serious and acute respiratory dysfunction. Despite improvements in treatment, ALI has high morbidity and mortality rates, particularly in the elderly [1]. The pathogenesis of ALI involves disruption of the alveolar capillary-epithelial barrier due to exaggerated pulmonary inflammation, increased permeability, and exudation of protein-rich serous fluid [2]. As a consequence, lung edema develops, and pulmonary gas exchange is suppressed [3]. Oxidative damage and the resulting activation of multiple signaling pathways is associated with the pathogenesis of ALI [4]. Intratracheal administration of lipopolysaccharide (LPS), a pathogenic endotoxin found in the outer membrane of Gram-negative bacteria [5], induces pulmonary inflammation by enhancing the production of reactive oxygen species (ROS) and activating inflammatory responses. LPS is therefore frequently used to induce ALI in animal models [6].

**Figure 1:**
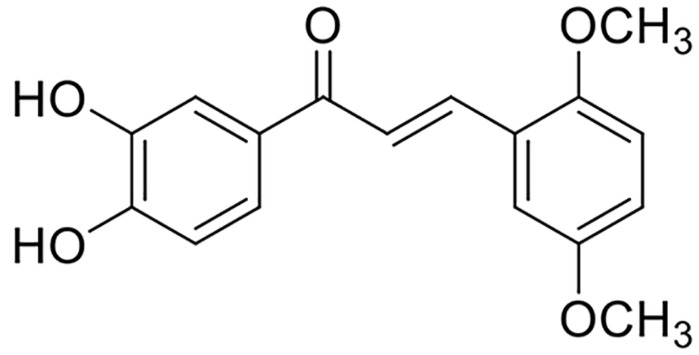
**Chemical structure of compound 33.**

Chalcones, a group of naturally occurring flavonoid compounds, exert antibacterial, antioxidant, anti-inflammatory, and anticancer effects [7, 8]. Previously, we synthesized several novel (*E*)-3,4-diphydroxychalcone derivatives and screened them for antioxidant activity. One of them, compound 33, exerted a particularly strong cytoprotective effect on hydrogen peroxide (H_2_O_2_)-induced oxidative damage *in vitro* and a neuroprotective effect against ischemia/reperfusion brain injury *in vivo* [9], its chemical structure is shown in Figure 1. However, whether compound 33 ameliorates LPS-induced inflammation and ALI is unknown.

LPS induces various intracellular signaling events, including activation of the mitogen-activated protein kinase (MAPK) pathway and nuclear factor κB (NF-κB) [10]. NF-κB, a rapid-acting pleiotropic transcription factor, induces overproduction of inflammatory mediators, such as tumor necrosis factor-α (TNF-α), interleukin-6 (IL-6), interleukin-1β (IL-1β), cyclooxygenase (COX2), and inducible nitric oxide synthase (iNOS) [11]. Activation of NF-κB is also implicated in the progression of LPS-induced ALI [12]. In addition, synthesis of the antioxidant enzyme heme oxygenase-1 (HO-1), which exerts a beneficial effect in rats with LPS-induced ALI, is regulated by nuclear factor erythroid-2-related factor 2 (Nrf2) [13].

In this study, we investigated the anti-inflammatory and antioxidant activity of compound 33 in LPS-challenged RAW 264.7 macrophages and in an animal model of LPS-induced ALI.

## RESULTS

### Compound 33 prevented LPS-induced ALI in vivo

**Figure 2:**
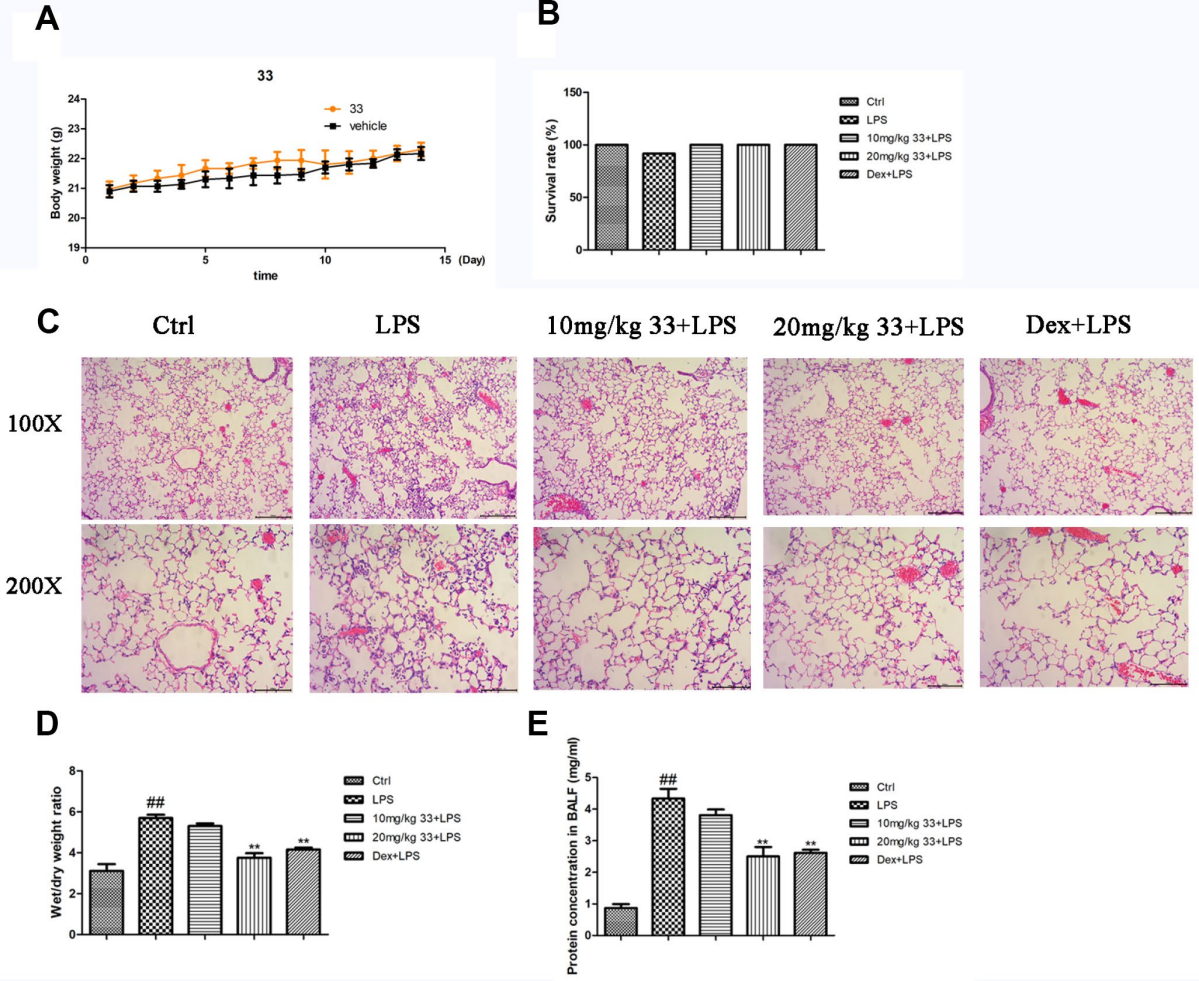
**Effects of compound 33 on mice with lipopolysaccharide (LPS)-induced acute lung injury (ALI).** (**A**) Mean body weights for the two groups over 15 days. (**B**) Survival rates of mice with LPS-induced ALI. (**C**) Lung tissues were stained with hematoxylin and eosin to show morphological changes. (**D**) Degree of tissue edema was evaluated using lung wet-to-dry weight ratios. (**E**) Total protein amounts in cell-free supernatant of bronchoalveolar lavage fluid (BALF). Data are presented as means ± standard error of the mean (SEM; n = 3–6). ^#^ P <0.05, ^##^ P <0.01, compared to the control group. * P <0.05, ** P <0.01, compared to the LPS group.

No mice died in compound 33 group and vehicle group in the toxicity experiment. Mean body weights were also similar between the two groups (Figure 2A), indicating that compound 33 is realtively safe for use in mouse models. Only one mouse died after 24h of LPS challenge, and survival rates did not differ significantly between the groups (Figure 2B). Compared to the control group, the lungs of mice challenged intratracheally with LPS exhibited thickening of the alveolar walls and interstitial spaces, disruption of endothelial and epithelial integrity, and neutrophil infiltration around the pulmonary blood vessels and airways (Figure 2C). However, pretreatment with compound 33 or dex prevented these LPS- induced pathological changes in the lungs. Moreover, pretreatment with compound 33 (20 mg/kg) or dex significantly reversed LPS-induced increases in lung wet weight (WW)/dry weight (DW) ratios and total protein concentration in the bronchoalveolar lavage fluid (BALF). More specifically, compound 33 reduced WW/DW ratio by 34% and protein levels by 42%. These effects were slightly stronger than those observed for dex, which reduced WW/DW ratio by 27% and protein amounts by 40% (Figure 2D, 2E). These results indicate that compound 33 ameliorated LPS-induced pathological changes in the lungs of ALI model mice.

### Compound 33 ameliorated LPS-induced inflammation and oxidative damage

**Figure 3:**
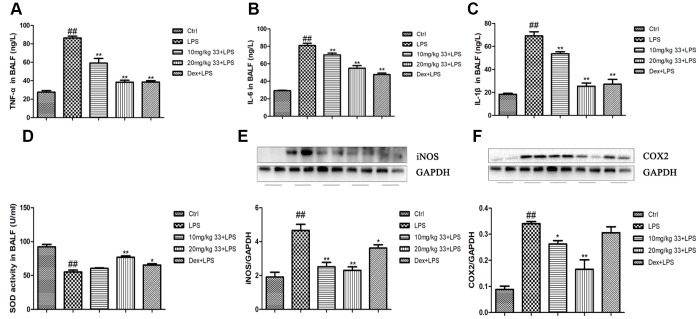
**Effects of compound 33 on LPS-induced inflammation and oxidative damage in mice.** (**A**–**C**) Levels of proinflammatory cytokines in cell-free supernatant of the BALF as determined using enzyme-linked immunosorbent assays. (**D**) Superoxide dismutase (SOD) activity in the BALF. Protein levels of the proinflammatory mediators (**E**) iNOS and (**F**) COX2 in the lungs. Data are presented as means ± SEM (n = 3–6). ^#^ P <0.05, ^##^ P <0.01, compared to the control group. * P <0.05, ** P <0.01, compared to the LPS group.

LPS stimulation markedly increased TNF-α, IL-6, and IL-1β levels in cell-free bronchoalveolar lavage fluid (BALF) supernatant compared to the control group. Pretreatment with compound 33 (10 or 20 mg/kg) or dex reversed the LPS-induced increases in the levels of these three proinflammatory cytokines (Figure 3A). More specifically, 20 mg/kg compound 33 reduced TNF-α levels by 56%, IL-6 levels by 32%, and IL-1β levels by 63%. Pretreatment with compound 33 prior to LPS administration also increased SOD activity in the BALF, which was significantly lower in the LPS group than in the control group (Figure 3D). Moreover, LPS markedly increased iNOS and COX-2 levels (Figure 3E, 3F), and compound 33 significantly reversed these increases. Thus, compound 33 exerts anti-inflammatory and antioxidant effects *in vivo.*

### Compound 33 inhibited the MAPK/NF-κB pathway and activated the Nrf2/HO-1 pathway in vivo

**Figure 4:**
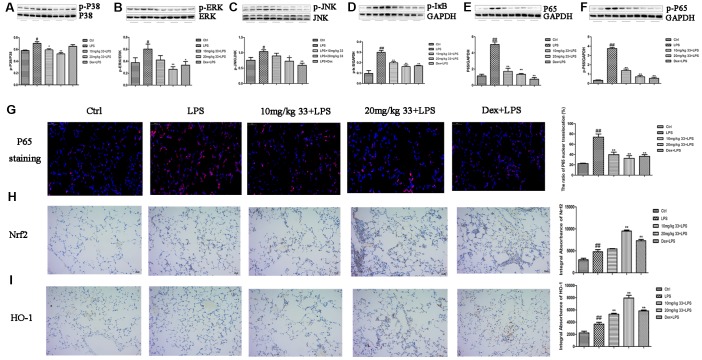
**Effects of compound 33 on the MAPK/NF-κB and Nrf2/HO-1 signaling pathways *in vivo*.** (**A**) p-P38, (**B**) p-ERK, (**C**) p-JNK, (**D**) p-IκBα, (**E**) P65, and (**F**) p-P65 protein levels relative to total P38, total ERK, total JNK, and GAPDH (loading control) were assayed using their respective antibodies. (**G**) Translocation of NF-κB P65 visualized by immunofluorescence staining. Immunohistochemical analysis of (**H**) Nrf2 and (**I**) HO-1 expression in lung tissue. Data are presented as means ± SEM (n = 3–6). ^#^ P <0.05, ^##^ P <0.01, compared to the control group. * P <0.05, ** P <0.01, compared to the LPS group.

Pretreatment with 20 mg/kg, but not 10 mg/kg, compound 33 reduced LPS-induced phosphorylation of P38, extracellular signal-regulated kinase (ERK), and c-Jun N-terminal kinase (JNK) (Figure 4A–4C). Furthermore, phosphorylated-IκBα (p-IκBα), P65, and p-P65 levels in lung tissues decreased in the presence of compound 33, especially for the 20 mg/kg dose (Figure 4D–4F). Immunofluorescence staining showed that nuclear NF-κB p65 levels in lung tissue increased upon exposure to LPS, and pretreatment with compound 33 blocked this nuclear translocation of NF-κB p65 (Figure 4G). Immunohistochemical analysis showed that Nrf2 levels were slightly higher in the LPS group than in the control group. Compound 33 also increased Nrf2 levels, and 20 mg/kg compound 33 increased Nrf2 levels more than LPS did (Figure 4H). HO-1 levels also increased after administration of compound 33 (Figure 4I). The MAPK/NF-κB and Nrf2/HO-1 pathways are therefore implicated in the effects of compound 33 on LPS-induced ALI *in vivo.*

### Compound 33 inhibited overproduction of proinflammatory markers and ROS

**Figure 5:**
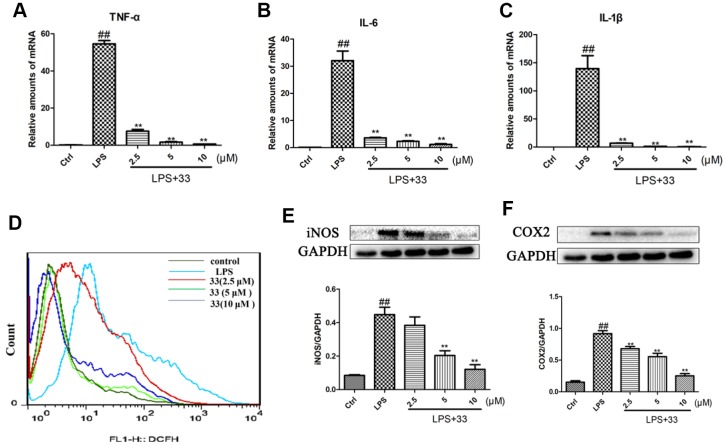
**Effects of compound 33 on LPS-induced increases in proinflammatory marker and reactive oxygen species (ROS) levels in RAW 264.7 cells.** RAW264.7 cells were stimulated with LPS or phosphate-buffered saline (PBS) for 12 h after pretreatment with 2.5, 5, or 10 μM compound 33. Total RNA was extracted and (**A**–**C**) proinflammatory cytokine mRNA levels were assayed. (**D**) Intracellular ROS level was assessed using loro-dihydro-fluorescein diacetate. (**E**) iNOS and (**F**) COX2 protein levels in RAW264.7 cells as determined using Western blotting. Data are presented as means ± SEM (n = 3–6). ^#^ P <0.05, ^##^ P <0.01, compared to the control group. * P <0.05, ** P <0.01, compared to the LPS group.

TNF-α, IL-6, and IL-1β mRNA levels increased markedly in RAW 264.7 cells challenged with LPS for 12h compared to vehicle-treated cells. Compound 33 reversed these LPS-induced cytokine level increases in a concentration-dependent manner, with 10 μM compound 33 exerting the most robust protective effect (Figure 5A–5C). Furthermore, the LPS-induced increase in iNOS and COX2 levels was also significantly reversed by compound 33 in a dose-dependent manner (Figure 5E, 5F). Finally, flow cytometry showed that pretreatment with compound 33 reversed the LPS-induced increase in intracellular ROS levels in a concentration-dependent manner (Figure 5D).

### Compound 33 inhibited the MAPK/NF-κB pathway and activated the Nrf2/HO-1 pathway in vitro

**Figure 6:**
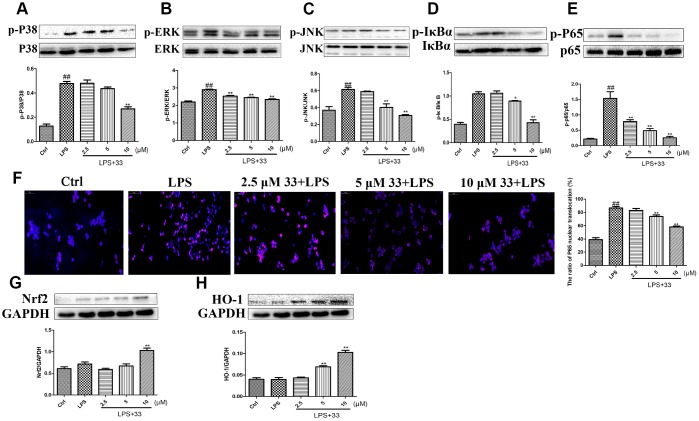
**Effects of compound 33 on the NF-κB and HO-1/Nrf2 signaling pathways *in vitro*.** RAW264.7 cells were treated with LPS for 1 h after pretreatment with 2.5, 5, or 10 μM compound 33. Protein levels were determined using Western blotting, and (**A**) p-P38/P38, (**B**) P-ERK/ERK, (**C**) p-JNK/JNK, (**D**) p-IκBα/IκBα, and (**E**) p-P65/P65 expression was quantified. (**F**) Nuclear translocation of NF-κB P65 visualized using immunofluorescence staining. Cells were pretreated with 2.5, 5, or 10 μM compound 33 or dimethyl sulfoxide for 1 h, and then exposed to LPS for 12 h. (**G**) Nrf2 and (**H**) HO-1 protein levels in cell lysates were analyzed; GAPDH was used as the control. Data are presented as means ± SEM (n = 3–6). ^#^ P <0.05, ^##^ P <0.01, compared to the control group. * P <0.05, ** P <0.01, for comparisons with the LPS group.

Western blotting showed that LPS significantly increased phosphorylation of P38, ERK, JNK, IκBα, and P65. Compound 33 reversed this effect, with 10 μM compound 33 exhibiting the highest potency (Figure 6A–6E). Immunofluorescence analysis revealed that LPS stimulation promoted nuclear translocation of NF-κB p65, and compound 33 markedly reversed this effect as well (Figure 6F). In addition, compound 33, but not LPS, increased Nrf2 and HO-1 protein levels (Figure 4G, 4H). These results suggest that the the MAPK/NF-κB and Nrf2/HO-1 pathways may mediate the protective effects of compound 33.

### Nrf2-siRNA transfection and Tin protoporphyrin IX (SnPP) reversed the anti-inflammatory effect of compound 33

**Figure 7:**
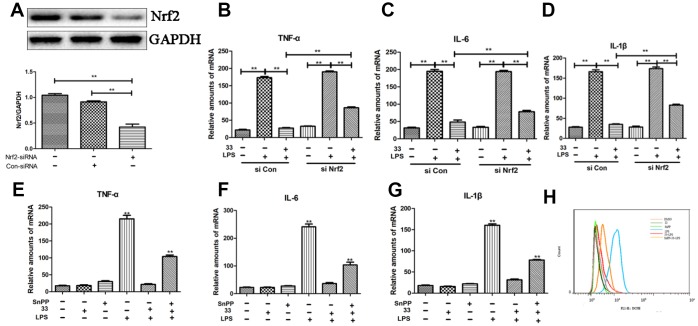
**Effects of Nrf2-siRNA transfection and tin protoporphyrin IX (SnPP) on compound 33-induced suppression of proinflammatory cytokine and ROS synthesis.** (**A**) Nrf2 protein levels were assayed using Western blotting after transfection with Nrf2-siRNA or Nrf2-negative control siRNA. Effects of Nrf2 silencing on compound 33-induced suppression of (**B**) TNF-α, (**C**) IL-6, and (**D**) IL-1β. To explore the function of the Nrf2/HO-1 signaling pathway in inflammatory responses and oxidative stress, SnPP, an inhibitor of HO-1, was administered to RAW264.7 cells; compound 33 was administered 1 h later. (**E**) TNF-α, (**F**) IL-6, and (**G**) IL-1β mRNA and (**H**) ROS levels were analyzed. * P <0.05, ** P <0.01, compared to the 33+ LPS group.

Western blot analysis showed that Nrf2 siRNA reduced Nrf2 levels compared to the negative control (Figure 7A). Nrf2 siRNA, but not control siRNA, also significantly reversed the compound-33-induced decrease in TNF-α, IL-6, and IL-1β levels (Figure 7B–7D). In addition, pretreatment with SnPP, an inhibitor of HO-1, blocked about 60–79% of the compound-33-induced reduction in IL-6, IL-1β, and TNF-α levels (Figure 7E–7G). Similarly, flow cytometry showed that SnPP increased ROS generation (Figure 7H). The Nrf2/HO-1 pathway therefore mediates the anti-inflammatory and antioxidant activities of compound 33.

## DISCUSSION

Inflammation facilitates ROS generation, which in turn promotes inflammatory reactions [14]. Inflammation and oxidative stress are linked biological processes that contribute to the pathogenesis of ALI [15, 16]. Intratracheal administration of LPS reportedly triggers a severe inflammatory response and oxidative damage by increasing the production of proinflammatory cytokines and ROS. We therefore used LPS to induce ALI in mice with histopathological characteristics similar to those of human ALI [6, 17]. ALI was induced in mice by intratracheal instillation of LPS (10 mg/kg) and was characterized by tissue edema, inflammatory cell infiltration, and disruption of the alveolar structure.

The low toxicity and diverse biological properties of natural and synthetic chalcones make them suitable candidates for drug discovery [18, 19]. We previously synthesized multiple chalcone derivatives and screened them for antioxidant potential. One of them, compound 33, exerted a significant protective effect in an animal model of middle cerebral artery occlusion and against H_2_O_2_-induced cellular injury *in vitro*. Compound 33 is therefore a candidate agent for the treatment of ischemic disorders [9]. However, relatively little was known about its anti-inflammatory activity and efficacy against LPS-induced ALI. Here, we report that intragastric administration of compound 33 ameliorated LPS-induced ALI, as indicated by histopathological features and decreased lung WW/DW ratios and total protein concentrations in BALF.

Inflammatory cells are activated in the early phase of ALI and lead to excessive production of proinflammatory cytokines (e.g., TNF-α, IL-6, and IL-1β). This results in the disruption of the alveolar epithelium, abnormal gas exchange, and a reduction in lung compliance [20]. IL-1β enhances the production of iNOS and COX2, which regulate the synthesis of nitric oxide and prostaglandin E2 [21]. Thus, inhibition of proinflammatory cytokine production is vital for the prevention of inflammatory reactions [22]. In this study, compound 33 significantly reduced TNF-α, IL-6, and IL-1β mRNA levels and inhibited the LPS-induced overproduction of these three proinflammatory cytokines in mice with ALI. Compound 33 also decreased iNOS and COX2 levels *in vivo* and *in vitro*. Notably, the effects exerted by compound 33 were equivalent to or greater than those of dex. Excessive activation and accumulation of inflammatory cells during ALI promotes the synthesis of proinflammatory factors and ROS. In this study, compound 33 suppressed the LPS-induced generation of ROS in RAW 264.7 cells. Compound 33 also upregulated SOD activity *in vivo*. Thus, compound 33 prevents LPS-induced oxidative damage by scavenging ROS. Taken together, these results indicate that compound 33 exhibits potent anti-inflammatory and antioxidant activity and attenuates LPS-induced tissue and cell damage.

Among the intracellular signaling pathways involved in inflammatory and immune responses, MAPK/NF-κB pathway might be particuarly important in mediating the effects of compound 33. Other chalcone analogues reportedly inhibit activation of the MAPK/NF-κB pathway *in vivo* and *in vitro* [7, 23]. The MAPK pathway plays a vital role in inflammation, and its activation is implicated in LPS-induced tissue injury, such as ALI [10]. Three subfamilies of MAPKs—P38, ERK, and JNK—are activated and phosphorylated in response to inflammatory stimuli such as LPS, as well as during LPS-induced ALI [4, 24]. Indeed, LPS stimulation markedly increased phosphorylation of P38, ERK, and JNK, and this effect was reversed by pretreatment with compound 33 *in vivo* and *in vitro*. NF-κB is a master transcription factor that plays a vital role in regulating the synthesis of proinflammatory markers during ALI [25]. Stimulants such as LPS activate the NF-κB pathway by promoting the phosphorylation and degradation of IκBα. This results in nuclear translocation of activated NF-κB, which then induces the transcription of genes encoding the proinflammatory cytokines iNOS and COX2 [26]. Our results suggest that compound 33 suppressed LPS-induced phosphorylation of NF-κB p65 and IκBα in RAW 264.7 cells. Moreover, compound 33 reversed the LPS-induced increase in nuclear translocation of NF-κB p65. These findings confirm that inhibition of the MAPK/NF-κB pathway is involved in the anti-inflammatory effects of compound 33.

The Nrf2 transcription factor regulates the expression of genes that encode antioxidant factors, including SOD and HO-1, by binding to antioxidant-response elements [27]. Activation of Nrf2/HO-1 results in antioxidant and antiapoptotic effects in many models of cell and tissue injury [28, 29]. In addition, the Nrf2/HO-1 signaling pathway was associated with the antioxidant effects of compound 33 after ischemia/reperfusion-related brain injury [9]. In this study, LPS significantly upregulated Nrf2 and HO-1 levels *in* *vivo* and *in* *vitro*, indicating that the Nrf2/HO-1 pathway was activated in response to LPS-induced injury. In addition, Nrf2 siRNA largely reveresed compound-33-induced decreases in TNF-α, IL-6, and IL-1β levels. Moreover, SnPP, an inhibitor of HO-1, significantly reversed the compound-33-induced suppression of proinflammatory cytokine and ROS synthesis, suggesting that the Nrf2/HO-1 pathway is functionally linked to the anti-inflammatory and anti-oxidative effects of compound 33.

**Figure 8:**
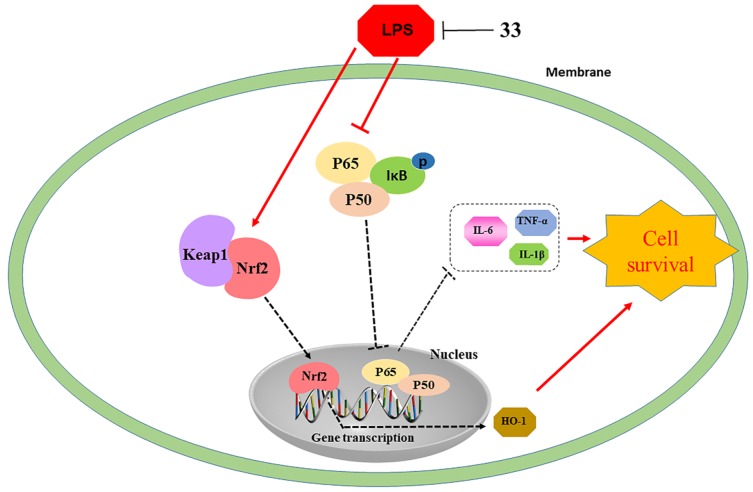
**Illustration of protection provided by compound 33 against LPS-induced lung injury.**

In conclusion, compound 33 protected against LPS-induced injury, inflammation, and oxidative damage *in* *vivo* and *in* *vitro*. The protective effects of compound 33 may be mediated by the inhibition of the MAPK/NF-κB pathway and activation of the Nrf2/HO-1 pathway (Figure 8).

## MATERIALS AND METHODS

### Reagents

Compound 33 with a purity of > 99% was synthesized; its chemical structure is shown in Figure 1. LPS (*Escherichia coli* O111:B4) was obtained from Sigma-Aldrich (St. Louis, MO, USA). ELISA detection kits were provided by Multisciences Biotech (Shanghai, China). The primers for TNF-α, IL-6, and IL-1β were purchased from Sangon Biotech Co., Ltd (Shanghai, China). The superoxide dismutase (SOD) and Bradford protein assay kits were purchased from Nanjing Jiancheng Bioengineering Institute (Nanjing, China). The ROS assay kit was obtained from Beyotime Biotechnology (Shanghai, China). Antibodies against iNOS, COX2, Nrf2, and HO-1 were purchased from Abcam (Shanghai, China), and antibodies against P38, p-P38, ERK, p-ERK, JNK, p-JNK, p-IκBα, IκBα, p-P65, P65, and glyceraldehyde-3-phosphate dehydrogenase (GAPDH) were purchased from Cell Signaling (Danvers, MA, USA). SnPP was obtained from Cayman Chemical (Ann Arbor, MI, USA). The control siRNA and Nrf2 siRNA were obtained from Shanghai GenePharma Co., Ltd. (Shanghai, China).

### Preparation of the mice

Animal experiments were conducted in accordance with the Guide for the Care and Use of Laboratory Animals of Wenzhou Medical University, Wenzhou, China, and the study protocol was approved by the University’s Institutional Animal Care and Use Committee (WYDW2017-0111). Male C57BL/6N mice (6–8 weeks of age and 20–24 g body weight) were housed in the Experimental Animal Center of Wenzhou Medical University and were fed standard laboratory chow and sterile water. ALI was induced after a 7-day acclimitization period [30].

### Toxicity evaluation of compound 33 in vivo

An acute toxicity experiment was performed to estimate the toxicity of compoud 33 [30]. Based on the methods of previous studies, 12 male C57BL/6N mice were randomly divided between the compound 33 and control groups and received a single 500 mg/kg dose of compound 33 or a similar dose of vehicle, respectively. Mortality rate and mouse body weights were then recorded for 15 days.

### LPS exposure and treatment

Based on previous reports [9, 31] and the results of preliminary experiments, mice were randomized into the following five groups: (i) vehicle-pretreated/phosphate-buffered saline (PBS)-exposed group (control group); (ii) vehicle-pretreated/LPS-exposed group (LPS group); (iii) compound 33-pretreated (10 mg/kg)/LPS-exposed group (10 mg/kg 33+LPS group); (iv) compound 33-pretreated (20 mg/kg)/LPS-exposed group (20 mg/kg 33+LPS group); and (v) dexamethasone (dex)-pretreated/ LPS-exposed group (dex+LPS group). Both compound 33 doses and 2 mg/kg dex were administered to the appropriate groups by gavage. Dex administration served as a positive control due to its efficacy against LPS-induced pulmonary inflammation. After 6 h, ALI was induced under anesthesia by intratracheal instillation of 10 mg/kg LPS; control group mice received an identical volume of PBS. After 24 h of LPS challenge, survival rates were recorded and surviving mice were euthanized using an approved protocol. Bronchoalveolar lavage fluid (BALF) was collected from the mice for analysis.

### Cell culture

Mouse RAW 264.7 macrophages (Cell Bank of the Chinese Academy of Science, Shanghai, China) were maintained in Dulbecco’s modified Eagle’s medium (DMEM) supplemented with 10% fetal bovine serum (FBS) and 100 U/mL penicillin-streptomycin and incubated at 37°C in an atmosphere with 5% carbon dioxide. RAW 264.7 cells were pretreated with 2.5, 5, or 10 μg/mL compound 33 and then exposed to 1 μg/mL LPS for 1 h. Next, the cells were harvested for analysis of MAPK/NF-κB pathway protein phosphorylation levels. After 12 h, RAW 264.7 cells were harvested for analysis of proinflammatory cytokine, ROS, nuclear NF-κB P65, and Nrf2/HO-1 pathway protein levels. To explore the role of the Nrf2/HO-1 pathway in the anti-inflammatory and antioxidant activity of compound 33, SnPP (20 μg/mL), an inhibitor of HO-1, was applied to RAW 264.7 cells 30 min before compound 33 was added.

### Assessment of lung histology

Lung tissues were dissected from the chest cavity, fixed in 10% neutral-buffered formalin for >24 h, dehydrated in an ethanol concentration gradient series, embedded in paraffin blocks, and cut into 4-μm-thick sections. The sections were stained with hematoxylin and eosin and examined under a light microscope (Olympus, Tokyo, Japan) in a blinded manner.

### Lung wet/dry weight ratio

After mice were euthanized, the lungs were harvested, washed with ice-cold PBS, blotted with filter paper, and wet weights (WW) were recorded. Lung tissues were then dehydrated in an oven at 60°C for 24 h and dry weights (DW) were recorded. The water content of the lung tissue was calculated as WW ÷ DW.

### Bronchoalveolar lavage fluid (BALF) analysis

Lungs were lavaged three times with 0.8 mL of ice-cold PBS. The resulting BALF was pooled and centrifuged at 12,000 rpm for 10 min. Levels of the proinflammatory cytokines TNF-α, IL-6, and IL-1β in the BALF were determined using commercially available enzyme-linked immunosorbent assay kits.

### Measurement of SOD activity

Lung tissues were excised from mice challenged with LPS for 24 h, homogenized, and lysed in extraction buffer. SOD activity in lung tissues was assayed using a commercially available kit.

### Extraction of total RNA and quantitative reverse transcriptase-polymerase chain reaction (qRT-PCR)

RAW 264.7 cells (2 × 10^6^) were cultured in 12-well plates and treated with the indicated concentrations of compound 33 prior to 12 h of LPS challenge. Next, total RNA was extracted using TRIzol reagent (Invitrogen, Carlsbad, CA, USA). The RNA was reverse-transcribed into cDNA using a HiScript II Q RT SuperMix for qPCR Kit (Vazyme, Nanjing, China). We also performed qRT-PCR using TB Green Premix ExTaq™ II (Takara Bio Inc., Shiga, Japan). The sequences of the forward and reverse primers were as follows: TNF: forward, 5′-GC GACGTGGAACTGGCAGAAG-3′, reverse, 5′-GCCAC AAGCAGGAATGAGAAGAGG-3′; IL-6: forward, 5′-TCCATCCAGTTGCCTTCTTG-3′, reverse, 5′-AAGTG CATCATCGTTGTTCATACA-3′; IL-1β: forward, 5′-A CTCCTTAGTCCTCGGCCA-3′, reverse, 5′-CCATCAG AGGCAAGGAGGAA-3′; and GAPDH: forward, 5′-AGGTCGGTGTGAACGGATTTG-3′, reverse, 5′-TGT AGACCATGTAGTTGAGGTCA-3′.

### Western blot analysis

Lung tissue lysates were generated by homogenizing lung tissues in tissue total protein lysis buffer (1 mM Tris-hydrochloride [HCl], 0.5 mM ethylenediaminetetraacetic acid [EDTA], 2 mM sodium chloride [NaCl], and 10% sodium dodecyl sulfate [SDS]). After stimulation with LPS for 1h or 12 h, RAW 264.7 cells were resuspended in extraction buffer (0.5 mM radioimmunoprecipitation assay buffer, 0.5 mM phenylmethylsulfonyl fluoride, and phosphatase inhibitors). Next, total tissue and cellular protein was extracted as described previously. Protein concentration was quantified using a bicinchoninic acid protein assay kit. Proteins were resolved using 10% or 12% SDS-polyacrylamide gel electrophoresis and transferred to a polyvinylidene difluoride membrane. After blocking in 5% dry milk in Tris-buffered saline with Tween (TBST) for 1.5 h, the membrane was incubated overnight in the presence of primary antibodies against GAPDH (1:1,000 dilution), iNOS (1:500), COX2 (1:1,000), Nrf2 (1:1,000), HO-1 (1:20,000), P38 (1:1,000), p-P38 (1:1,000), ERK (1:1,000), p-ERK (1:1,000), JNK (1:1,000), p-JNK (1:1,000), p-P65 (1:500), P65 (1:500), p-IκB (1:1,000), and IκB (1:1,000). The membrane was then washed in TBST and incubated with the appropriate horseradish peroxidase-conjugated secondary antibody for 1 h at room temperature. Immunoreactive bands were visualized using chemiluminescence (ECL) and quantified by densitometry using ImageLab software (ver.6.0). Bio-Rad Laboratories, Hercules, CA, USA). Protein levels were normalized to that of GAPDH*.*

### Immunohistochemical analysis

Lung-tissue sections were dewaxed and hydrated. Nonspecific antigen sites were blocked using normal goat serum, and the sections were incubated with anti-Nrf2 and -HO-1 primary antibodies overnight at 4°C. Next, the sections were washed with PBS and immunostained with 50 μL of biotin-conjugated secondary antibody for 1 h at room temperature. Signals were visualized using 3,3′-diaminobenzidine tetrahydrochloride hydrate (DAB), and the sections were stained with hematoxylin, mounted, and observed under a light microscope. Nrf2- and HO-1–positive cells were counted in five random fields at 400× magnification. Images were analyzed using Image-Pro Plus software (v. 6.0; Media Cybernetics, Rockville, MD, USA).

### Immunofluorescence staining of P65

Following deparaffinization using xylene and dehydration in a graded alcohol series, lung sections were incubated with 3% H_2_O_2_ for 10 min and blocked in bovine serum albumin (BSA) for 1 h. After pretreatment with compound 33 and 12 h of exposure to LPS, RAW 264.7 cells were fixed in paraformaldehyde for 45 min and then placed in 5% BSA in PBS for 30 min. The sections or cells were incubated in the presence of the anti-p65 primary antibody (1:200 dilution) overnight at 4°C, and then incubated with the phycoerythrin-labeled secondary antibody (1:400) for 1 h at room temperature. Nuclei were visualized using 4′,6-diamidino-2-phenylindole (DAPI). Finally, the sections/cells were visualized under a fluorescence microscope at 400× magnification (Nikon, Tokyo, Japan).

### Cell transfection

RAW 264.7 cells (2 × 10^6^) were grown in six-well plates and were transiently transfected with Nrf2-negative control siRNA or Nrf2-siRNA using Lipofectamine 2000 (Thermo Scientific, Shanghai, China) for 72 h upon reaching 50-60% confluence. Transfection was confirmed using Western blot analysis.

### Measurement of ROS levels in vitro

RAW 264.7 cells (2 × 10^6^/well) were grown in six-well plates pretreated with the indicated doses of compound 33, and then exposed to LPS (100 ng/mL) for 12 h. The cells were harvested and incubated with 1 mL of FBS-free DMEM containing 1 μL of loro-dihydro-fluorescein diacetate for 20 min at 37°C. After washing three times with PBS, ROS levels were assayed using flow cytometry (FACSCalibur; BD, Franklin Lakes, NJ, USA), and the results were analyzed using FlowJo software (v. 10.5.3; Ashland, OR, USA).

### Statistical analyses

Data were analyzed using SPSS software (v. 19.0; IBM Corporation, Armonk, NY, USA) and are expressed as means ± standard error of the mean (SEM). The significance of differences among samples was assessed using the Mann–Whitney test. Images were digitally processed using Prism (v. 5; GraphPad, La Jolla, CA, USA) and Photoshop software (ver. 5.0; Adobe Inc., Mountain View, CA, USA). A value of *P* <0.05 indicated a statistically significant difference.
